# Adipokines, Myokines, and Hepatokines: Crosstalk and Metabolic Repercussions

**DOI:** 10.3390/ijms22052639

**Published:** 2021-03-05

**Authors:** Ana Rita de Oliveira dos Santos, Bárbara de Oliveira Zanuso, Vitor Fernando Bordin Miola, Sandra Maria Barbalho, Patrícia C. Santos Bueno, Uri Adrian Prync Flato, Claudia Rucco P. Detregiachi, Daniela Vieira Buchaim, Rogério Leone Buchaim, Ricardo José Tofano, Claudemir Gregório Mendes, Viviane Alessandra Capelluppi Tofano, Jesselina F. dos Santos Haber

**Affiliations:** 1 Department of Biochemistry and Pharmacology, School of Medicine, University of Marília (UNIMAR), Avenida Higino Muzzi Filho 1001, Marília 17525-902, São Paulo, Brazil; anaritasantos@hotmail.com (A.R.d.O.d.S.); barbarazanuzo@hotmail.com (B.d.O.Z.); vitormiola@hotmail.com (V.F.B.M.); patriciabueno@hotmail.com (P.C.S.B.); uriflato@hotmail.com (U.A.P.F.); danibuchaim@hotmail.com (D.V.B.); ricardotofano@hotmail.com (R.J.T.); claudemirmendes@hotmail.com (C.G.M.); vivianetofano@gmail.com (V.A.C.T.); jesselinahaber@hotmail.com (J.F.d.S.H.); 2 Postgraduate Program in Structural and Functional Interactions in Rehabilitation, University of Marilia (UNIMAR), Avenida Hygino Muzzy Filho 1001, Marília 17525-902, São Paulo, Brazil; claudiarucco@gmail.com; 3 Department of Biochemistry and Nutrition, Faculty of Food Technology of Marília, Marília 17500-000, São Paulo, Brazil; 4 Department of Animal Sciences, School of Veterinary Medicine, University of Marília (UNIMAR), Avenida Higino Muzzi Filho 1001, Marília 17525-902, São Paulo, Brazil; 5 Medical School, University Center of Adamantina (UniFAI), Adamantina 17800-000, São Paulo, Brazil; 6 Department of Biological Sciences, Bauru School of Dentistry, University of São Paulo (FOB–USP), Alameda Doutor Octávio Pinheiro Brisolla 9-75, Bauru 17040, São Paulo, Brazil; rogerioLeone@hotmail.com

**Keywords:** adipokines, myokines, hepatokines, metabolism, cardiovascular diseases

## Abstract

Adipose, skeletal, and hepatic muscle tissues are the main endocrine organs that produce adipokines, myokines, and hepatokines. These biomarkers can be harmful or beneficial to an organism and still perform crosstalk, acting through the endocrine, paracrine, and autocrine pathways. This study aims to review the crosstalk between adipokines, myokines, and hepatokines. Far beyond understanding the actions of each biomarker alone, it is important to underline that these cytokines act together in the body, resulting in a complex network of actions in different tissues, which may have beneficial or non-beneficial effects on the genesis of various physiological disorders and their respective outcomes, such as type 2 diabetes mellitus (DM2), obesity, metabolic syndrome, and cardiovascular diseases (CVD). Overweight individuals secrete more pro-inflammatory adipokines than those of a healthy weight, leading to an impaired immune response and greater susceptibility to inflammatory and infectious diseases. Myostatin is elevated in pro-inflammatory environments, sharing space with pro-inflammatory organokines, such as tumor necrosis factor-alpha (TNF-α), interleukin-1 (IL-1), resistin, and chemerin. Fibroblast growth factor FGF21 acts as a beta-oxidation regulator and decreases lipogenesis in the liver. The crosstalk mentioned above can interfere with homeostatic disorders and can play a role as a potential therapeutic target that can assist in the methods of diagnosing metabolic syndrome and CVD.

## 1. Introduction

Eating behavior, delimited by cultural and social aspects, has a substantial impact on health conditions and the development of cardiovascular diseases and inflammatory complications [1]. The popularity of cheap, high-calorie foods associated with sedentary living, high workload, and time constraints has contributed substantially to the global increase in obese individuals. The excess weight combined with hyperglycemia predisposes oxidative stress and inflammation [2] can impair insulin signaling and promote the development of comorbidities, such as type 2 diabetes mellitus (DM2), hypertension, and other factors that induce cardiovascular complications. [2]. In accordance with the International Obesity Task Force, an estimated 1.7 billion people are vulnerable to health risks determined by body weight. Moreover, 2.5 million deaths annually are related to the increase in the body mass index (BMI), which is expected to double by 2030 [3].

These changes in lifestyle habits have allowed cardiovascular disease (CVD), especially coronary artery disease, stroke, and heart failure, to occupy the number one position among the leading causes of death worldwide today [4,5]. According to the World Health Organization (WHO), CVDs are responsible for approximately 17.9 million deaths per year, corresponding to 31% of total deaths worldwide in recent decades, in addition to being responsible for increased morbidity and lifelong disability [6]. The increases in these mortality and morbidity rates are interpreted as a trend in developed and developing countries due to the difficulty in modifying the consolidated lifestyle habits and the several associated comorbidities that hinder the prognosis and perpetuate cardiovascular risks. From this perspective, CVDs are real burdens for health systems due to their severity, prevalence, and difficulty to treat [7].

In contrast, technological advances in medicine have raised quality and life expectancy, giving light to another trend in the contemporary world: population aging. However, physiological changes inherent to the aging process, such as sarcopenia, decreased cardiovascular, and cognitive function, added to the population’s bad habits, seem to predispose the body to more significant cardiovascular risks and other chronic diseases associated with aging [8]. Therefore, physical activity and good eating habits must be encouraged to achieve healthy aging. Otherwise, without a doubt, such situations tend to compromise further the overload of health systems and the daily challenge of professionals [9].

In the molecular context, organokines are increasingly investigated because they are related to metabolism. Adipose, skeletal, and hepatic muscle tissues are the main endocrine organs that produce adipokines, myokines, and hepatokines. These biomarkers can be harmful or beneficial to the organism and still perform crosstalk, acting through the endocrine, paracrine, and autocrine pathways. Therefore, they have specific associations with insulin resistance, diabetes mellitus, obesity, metabolic syndrome, and CVD [10]. Thus, adipokines, myokines, and hepatokines may play, in the near future, roles of new markers for diagnosis and prognosis, elucidating the mechanisms involved in metabolic disorders and CVD, thereby facilitating innovative therapeutic approaches [11].

The gathering of knowledge about adipokines, myokines and hepatokines and the cross-talk between them, brings to light the understanding of how lifestyle changes leading to obesity and its metabolic consequences, results in marked changes in the secretion profile of these substances, which may be the basis of many disorders. Moreover, many organokines are secreted by all three tissues. Therefore, understanding the mechanisms involved in the secretory pattern can be useful in the investigation of many diseases.

Given the above, this study aims to review the crosstalk among adipokines, myokines, and hepatokines to contribute to the study of future diagnostic methods and therapeutic targets in managing cardiovascular diseases and metabolic disorders. To the best of our knowledge, no review has shown a comprehensive overview of crosstalk or these organokines with focus on insulin resistance, diabetes, metabolic syndrome, and CVD. An integrated approach related to organokines are needed to developed new treatments for obesity, diabetes, metabolic syndrome and CVD.

## 2. Discussion

### 2.1. Adipokines

Adipokines are molecules released by adipose tissue through endocrine pathways, capable of controlling lipid metabolism and interfering with insulin sensitivity, appetite, fibrogenesis, and liver fat deposition. Leptin and adiponectin (Table 1) are the classic adipokines of adipose tissue and have a substantial relationship in the pathogenesis of obesity and metabolic complications [12]. Adipose tissue responds to excess energy through energy storage by increasing adipocytes. In obesity, its hypertrophy is directly related to chronic low-grade inflammation and an increase in chemotactic molecules, in addition to a reduction in adiponectin levels and the onset of leptin resistance. With the increase in adipose tissue, mainly in the visceral region, there is a change in the expression pattern of M1 macrophages that are related to gene modulation and the consequent increase in the release of pro-inflammatory mediators, such as leptin (there will be resistance to the action of leptin), resistin, tumor necrosis factor-alpha (TNF-α), interleukin (IL)-6, IL-18, plasminogen activator inhibitor (PAI-1), and reduction in anti-inflammatory mediators such as IL-10 [10,11,13]. Table 1 shows the main characteristics of classic adipokines in adipose tissue.

**Figure 1:**
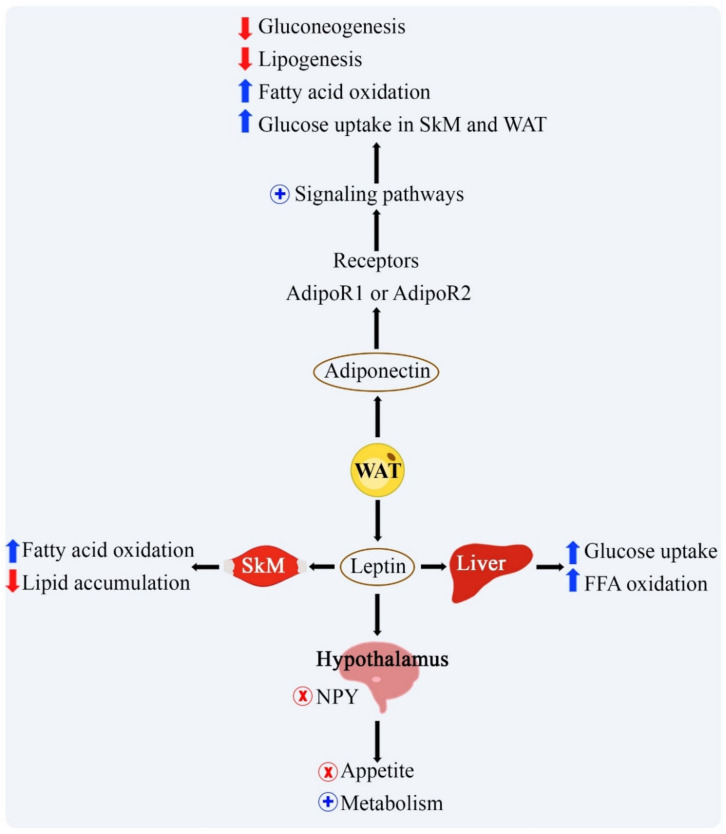
Effects of leptin and adiponectin on the metabolism of the central nervous system, skeletal muscle, and liver. Black arrows represent the flow in metabolism; blue arrow represents increased action; red arrow represents decreased action; + represents activation; x represents inhibition. FFA: free fatty acid; NPY: neuropeptide Y; SkM: skeletal muscle; WAT: white adipose tissue.

Leptin is released by subcutaneous white adipose tissue (WAT) [39] and acts as an endocrine signal by reducing appetite and stimulating energy expenditure by activating pro-opiomelanocortin (POMC)—expressing neurons, inhibiting the hypothalamus agouti-related protein (AgRP) and neuropeptide Y (NPY). In addition, leptin increases the oxidation and absorption of glucose and free fatty acid (FFA) in skeletal muscles and promotes intrahepatic lipid reduction through fatty acid oxidation (Figure 1). In obesity, the increase in leptin levels is associated with the state of leptin resistance, in addition to the increase in reactive oxygen species (ROS), IL-6, TNF-α, and chemokines. Hyperleptinemia contributes to the action of pro-inflammatory cytokines and continuous infiltration of M1 macrophages [11]. Leptin stimulates the division of hepatic stellate cells and the synthesis of pro-fibrogenic and pro-inflammatory factors in these cells; moreover, it induces the synthesis of transforming growth factor b (TGFβ) in Kupffer cells [40]. Elevated levels of leptin were associated with development, progression and prognostication of non-alcoholic fatty liver disease (NAFLD) [41]. Metreleptin is a recombinant methyl-human leptin hormone showing efficacy in children with congenital leptin deficiency reversal of obesity and metabolic improvement [42]. In adults with hyperleptinemia and leptin resistance administration of metreleptin showed marginal benefits with a reduction in glycated hemoglobin [43].

Adiponectin, an antisteatotic, anti-inflammatory, and antifibrotic adipokine, is expressed by adipocytes from WAT and brown adipose tissue (BAT). It acts on insulin sensitivity, and its plasma reduction is related to insulin resistance and glucose intolerance, predisposing to advanced liver injury [44]. Plasma levels of adiponectin can be reduced with the increase in pro-inflammatory cytokines such as TNF-α and IL-6, present in obesity, in addition to the levels of myostatin, a myokine that can elevate the adipose tissue mass [45]. This adipokine contributes significantly to anti-inflammatory action, thermogenesis, lipolysis, and oxidation of fatty acids in skeletal muscle and liver. Its action begins with the binding to its AdipoR1 or 2 receptors and the activation of AMP-activated protein kinase (AMPK), leading to reduced lipogenesis and gluconeogenesis and increased glucose absorption by skeletal muscle and WAT [46]. Recently, an orally active synthetic adiponectin receptor agonist, AdipoRon, was developed and studied in an experimental animal study (mice) showing improvement in vascular function in the mesenteric arteries independently of endothelium-mechanism [47]. Figure 1 shows some effects of the main adipokines.

In addition to the classic adipokines, the adipose tissue produces other adipokines in different circumstances. Resistin, a mediator of obesity-related insulin resistance, is a pro-inflammatory adipokine correlated with fat mass that acts on endothelial dysfunction and cardiovascular diseases [15]. In humans, it is produced by macrophages and monocytes infiltrated in adipose tissue. In glucose metabolism, resistin is related to activating an inhibitor of insulin signaling, the suppressor of cytokine signaling 3 (SOCS3). In the same perspective, IL-6, in adipose tissue, has pro-inflammatory characteristics, being directly related to the BMI, and is augmented in individuals with insulin resistance, obesity, and DM2. It is produced by macrophages and stimulated by the activation of the nuclear factor κ-light-chain-enhancer of activated B cells (NF-κB) and acts in inhibiting the expression of insulin receptor substrate 1 (IRS1) and glucose transporter type 4 (GLUT4) in adipocytes [14].

Asprosin, which is encoded by the *FBN1* locus, has a role in the mediation of the lipodystrophy phenotype [48]. It is an orexigenic hormone induced by fasting and produced by WAT, and increases food consumption and body weight through Agouti-related protein neurons (AgRP), and accelerates the production of liver glucose, activating the G protein-cAMP-protein kinase A (PKA) pathway [16,17]. In a clinical study, it was found that obese people have a high concentration of asprosin, and it is also closely related to insulin resistance in humans, which is proven when higher serum levels of asprosin are identified in patients with DM2 [49]. Asprosin levels are elevated in comorbidities such as obesity and DM2. Gozel and Klink [50] showed that the levels of aprosin in saliva are significantly increased in newly diagnosed DM2 patients, and the authors suggested that this organokine might be an important risk factor related to the development of diabetes. For these reasons, asprosin can be an important biomarker for predicting the development of diabetes. Another interesting study showed that asprosin is associated with the modulation of cardiac mitochondrial functions and in patients with dilated cardiomyopathy patients, elevated asprosin level is associated with less adverse cardiovascular issues in five years. These protective actions of asprosin can be associated to its functions of enhancing mitochondrial respiration under hypoxia [51]. On the other hand, the adipokine named chemerin, which is secreted and an inactive molecule, is activated by inflammatory and coagulation serine proteases, accentuates glucose intolerance, and makes insulin signaling difficult. Its action occurs through the chemerin receptor (ChemR) 23, and its receptors are mostly concentrated in adipose tissue and hepatocytes, despite existing throughout the human body [18].

In turn, omentin acts to improve insulin sensitivity and glucose absorption and acts as an anti-atherosclerotic factor. The main circulating form of omentin is omentin-1, known as interleukin-1, expressed in visceral tissue adipose, and its levels are regulated by fibroblast growth factor-21, dexamethasone, glucose, and insulin [19]. Still, studies suggest that asprosin may be a viable marker for unstable angina pectoris (UAP), and the degree of severity of acute coronary syndrome (SCA) with UAP can be predicted by this molecule [52].

Another substance recently introduced as an adipokine is fibroblast growth factor 21 (FGF21), whose exposure to cold or physical exercise causes its expression in white and BAT. In humans, FGF21 is responsible for the browning of WAT, activation of brown adipocytes, and lipolysis. In addition, it increases the expression of uncoupling protein 1 (UCP1) and other thermogenic genes and stimulates the expression of adiponectin in the bloodstream and WAT [20].

The secreted frizzled-related protein 5 (SFRP5), recently identified as an adipokine, is expressed in WAT in higher amounts compared to other tissues. SFRP5 is associated with comorbidities such as obesity, DM2, insulin resistance, and atherosclerosis, inhibiting the wingless-type family member 5a signaling (Wnt5a) and the non-canonical Wnt family and regulating the expression of pro-inflammatory cytokines [14,21].

Lipocalin 2 (LCN2), also associated with neutrophil gelatinase-associated lipocalin (NGAL), is a glycoprotein identified as the cytokine of adipose tissue present in low-level systemic inflammation in obese patients with metabolic syndrome. Its receptors, the megalin/glycoprotein GP330 and SLC22A17 or 24p3R, the solute carrier family 22 members 17 binding mouse LCN2, are involved in regulating inflammation and the transport of fatty acids and iron [23]. High levels of LCN2 are associated with CVD, vascular remodeling, and instability of atherosclerotic plaques by regulating the metalloproteinase activity (MMP-9) [22].

In the same perspective, visceral adipose tissue-derived serpin (vaspin), a protein that inhibits serine protease, is also found in greater quantities in obese individuals with DM2 and expressed by adipose tissue and skeletal muscle [53]. However, the function of vaspin is to improve glucose intolerance and insulin sensitivity and reduce the synthesis of pro-inflammatory cytokines [24]. Studies show that vaspin protects vascular tissues from fatty acid-induced apoptosis via the signaling pathway PI3K/Akt/eNOS [25]. It can also suppress vascular cell proliferation and migration by reducing the c-Jun N-terminal kinase (JNK) and ERK1/2 pathways and increasing collagen via the PI3K/Akt pathways [54].

Follistatin-like 1 (FSTL1), an adipokine and myokine reported as a pre-adipocyte/adipocyte-secreted protein, has a pro-inflammatory action and possible relationship with overweight and obesity. In the state of low-grade chronic inflammation, there is a greater number of pre-adipocytes that express FSLT1; however, morbidity and super obesity are associated with a decline in FSTL1 due to loss of adipogenesis, increased maturated adipocytes, cellular senescence, and anti-apoptotic reduction in FSLT1 [26].

The secreted acidic protein rich in cysteine (SPARC), known as BM40 or osteonectin, modulates the expression of pro-inflammatory cytokines involved in insulin resistance and adipogenesis [23]. Although initially found in bone tissue, SPARC is expressed in most tissues, being one of the proteins in the extracellular matrix of adipose tissue. Its function is related to the inhibition in the initial stage of adipogenesis through inhibiting the mitotic clonal expansion of pre-adipocytes [27]. Therefore, the inhibition of adipogenesis can restrict the storage of triglycerides in tissue adipose, increasing circulating levels (systemic hyperlipidemia) and lipid deposition in the liver and skeletal muscle [55].

C1q/TNF-related proteins (CTRPs) are related to a family of adipokines with a structure similar to adiponectin, involved in the regulation of glucose and fat metabolism in peripheral tissues, food intake, and inflammatory processes of adipose tissue [28]. Its action occurs through several receptors, including adipoR1 and adipoR2. Studies in mice have demonstrated that the expression of CTRP6, regulated for adipose tissue in obesity and diabetes, is secreted by stromal vascular cells and macrophages, and its increased expression substantially compromised the disposal of glucose in peripheral tissues [23]. On the other hand, the family with sequence similarity to 19 member A5 (FAM19A5), initially demonstrated in mice, is released by adipose tissue, and its expression is reduced in obesity. Studies demonstrate that FAM19A5 inhibits vascular smooth muscle cell proliferation and inflammation related to cardiovascular disease through obesity [29].

From the same perspective, the wingless-type inducible signaling pathway protein-1 (WISP1), an adipokine associated with the extracellular matrix, is expressed for visceral and subcutaneous tissue adipose and stimulates the cytokine response in macrophages associated with tissue adipose. Experimental studies of visceral obesity have found that WISP1 can induce mesenchymal stem cells’ proliferation and, consequently, the expansion of tissue adipose [30]. On the other hand, the glycoprotein progranulin, secreted by adipocytes from visceral tissue adipose, macrophages, chondrocytes, and other cells, has anti-inflammatory properties due to competition with TNF-α receptors (TNFR1 and TNFR2). However, hyperprogranulinemia present in obesity is associated with insulin resistance and deficient insulin signaling, whereas its deficiency can protect from diet-induced insulin resistance [31,32]. Nesfatin-1, in turn, involved in energy homeostasis and satiety induction, is described as an anorexigenic molecule secreted by subcutaneous adipose tissue, pancreatic cells, gastric mucosa, and hypothalamus. In rodents, injections of nesfatin-1 inhibit food intake, and its lack in the brain causes an increase in appetite, fat, and body weight. In addition, nesfatin-1 can regulate gastric distension and motility via the melanocortin pathway in the central nucleus of the amygdala [23].

Another adipokine secreted by adipose tissue is visfatin, a class type II phosphoribosyltransferase homodimer, which increases in obese people. Its secretion is more significant in the subcutaneous adipose tissue and is associated with an enzymatic activity such as nicotinamide phosphoribosyltransferase (NAMPT) [33]. Visfatin in high concentrations causes insulin resistance and pancreatic beta-cell dysfunction and produces inflammation in adipocytes [34]. Fetuin-A (Fet-A), classically considered a hepatokine, is a natural insulin inhibitor tyrosine kinase and is associated with insulin resistance and inflammation in rodents and humans. Fet-A levels are increased in obese individuals with diabetes [35]. On the other hand, the adipokine zinc-α2-glycoprotein (ZAG) is expressed by both white and BAT and directly accelerates lipid metabolism [38]. Its action is in regulating lipogenesis and lipolysis enzymes, in the stimulation of adiponectin production, and the browning of WAT. Studies show that serum ZAG levels are lower in overweight or obese patients [37].

### 2.2. Myokines

In addition to playing important roles as a reservoir and consumer of energy and a leading role in carbohydrate metabolism, skeletal muscle tissue (SkM) has been associated with substantial secretory functions in recent studies. These secretion products, called myokines, are peptides, cytokines, or growth factors that show a diversity of autocrine, paracrine, or endocrine actions. These molecules are expressed mainly in physical activities or absence, allowing its communication with other body tissues, thereby showing actions that can be considered beneficial for most myokines or related to metabolic dysfunctions [8,14]. A vast number of myokines were identified, although other tissues also produce some. Each myokine appears to be related to a specific type of muscle fiber and different physical exercise modalities [56] (Table 2).

Irisin, one of the most recent myokines identified, is a cleavage product of type III transmembrane fibronectin protein containing domain 5 (FNDC5), released after cleavage of the carboxy group. It is a transmembrane protein with a type III fibronectin domain secreted after physical exercise, which has shown participation in the browning of white tissue adipose (WAT) process by increasing the expression of uncoupling protein-1, the respiratory chain of BAT, which promotes energy expenditure especially in the form of heat, contributing to weight loss [11]. Therefore, induction of adipocyte browning by irisin suppresses adipogenesis and cholesterol synthesis and optimizes lipid oxidation and lipidic homeostasis by extension. In addition, irisin appears to increase insulin sensitivity, promoting glucose transporter mobilization in insulin-dependent tissues, and improves metabolic syndrome and CVD. Studies point to a relationship between high levels of irisin and low atherosclerosis burden in coronary arteries, proving its cardioprotective effects [8].

Myostatin exhibits negative consequences when it is at increased levels, as it acts as a facilitator of muscle mass loss, either by inhibiting factors that promote tissue growth or by stimulating degradation mechanisms. This reduction in muscle mass, mediated by myostatin, facilitates the appearance of metabolic disorders, such as resistance to insulin and accumulation of fat in the liver, since skeletal muscle is the main source of glucose-dependent glucose uptake. Unlike most myokines, exercise seems to decrease myostatin expression. Possibly, myostatin inhibition may be a therapeutic target for muscle mass loss disorders, such as atrophy, sarcopenia, and cancer [45]. In contrast, there is follistatin, a myokine, and hepatokine, released in the context of physical activity, which is opposed to myostatin’s actions, directly inhibiting it through binding. Thus, follistatin contributes to the increase in muscle mass, optimizing glucose uptake by SkM and WAT lipolysis [11,13].

Fibroblast growth factor 21 (FGF21) is produced by several tissues classified as myokine, adipokines, and hepatokine. Elevated levels of FGF21 after exercise reveal crucial functions for glucose and lipid metabolism. It increases insulin sensitivity, reduces plasma glucose levels, and has lipolytic activity, which decreases plasma triglyceride levels [10]. It has great therapeutic potential in several metabolic comorbidities, such as obesity, DM2, and metabolic syndrome. Despite this, the clinical study by Mikolajczak et al. showed that FGF21 levels are increased in the state of obesity, possibly as an adaptive mechanism against insulin resistance and its consequences [57].

Apelin, released mainly in resistance exercises, has an important anti-inflammatory role and other essential actions in metabolism, control of cardiac muscles and blood pressure, new vessels’ formation, and control of cell cycle and death. The decrease in plasma apelin is commonly observed in the elderly due to the reduction in muscle mass. Therefore, this myokine is also pointed out as a biomarker of early sarcopenia [58]. Furthermore, released in resistance modalities, myonectin acts on the metabolism of lipids, increasing their uptake by adipose tissue and liver, thus decreasing the plasma concentration of FFA. A drop in myonectin levels, which can be caused by physical inactivity or a high-fat diet, is related to excess circulating FFA and subsequent accumulation in other tissues and insulin resistance [20]. This myokine shows positive effects on the cardiovascular system, which may reveal a potential therapeutic target in CVD treatment [8].

Interleukin-6 (IL-6), the first myokine to be described, is often characterized as a pro-inflammatory cytokine produced by various tissues, being considered myokines, adipokines, and hepatokine. Despite the pro-inflammatory cytokine label, mainly due to their role as an adipokine, IL-6 levels are increased after physical exercises, which promotes the secretion of interleukin-10 (IL-10), an IL-1 antagonist and possible inhibitor of TNF-α, characterizing an anti-inflammatory environment after increasing the concentration of IL-6 released by muscle tissue. It also stimulates insulin release by increasing the expression of the glucagon-1-like peptide (GLP-1) [11,59]. Moreover, some experimental studies point to a possible correlation between increased levels of IL-6 and inhibition of appetite [52].

IL-15 has anti-inflammatory properties, mainly by inhibiting the expression of TNF-α, which plays a role in oxidative stress. It also prevents the reduction in muscle mass and increases the uptake of glucose by skeletal muscle by stimulating the mobilization of glucose transporters 4 (GLUT 4), contributing to muscle hypertrophy [18]. Additionally, it helps in the reduction in visceral adipose tissue without reducing subcutaneous fat, showing performance in muscle-fat crosstalk [14].

SPARC is released in episodes of resistance exercises and muscle hypertrophy and has also been related to the optimization of glucose uptake by increased expression and translocation of GLUT 4 by activating the AMPK signaling pathway [56]. It is also able to inhibit the formation of adipose tissue, increase the release of insulin and promote erythropoiesis [14]. Some studies address the role of SPARC in activating caspase-3 and caspase-8 dependent apoptosis, which reveals a potential benefit of physical exercise in decreasing the incidence of colon cancer [60].

The brain-derived neurotrophic factor (BDNF) is also released by muscle and brain tissue after exercise; however, unlike other myokines, it is not released into the bloodstream [61]. The study performed by Pedersen [56] suggests that exercise stimulates the release of myokines, such as cathepsin B and irisin, which in turn cross the blood–brain barrier to induce cerebral BDNF secretion. This acts primarily on neurons through tyrosine receptor kinases (Trk), allowing their growth and survival and playing a fundamental role for memory and learning. Besides that, it is also related to glucose and lipid metabolism regulation and increased insulin sensitivity when released by muscle [14].

**Figure 2:**
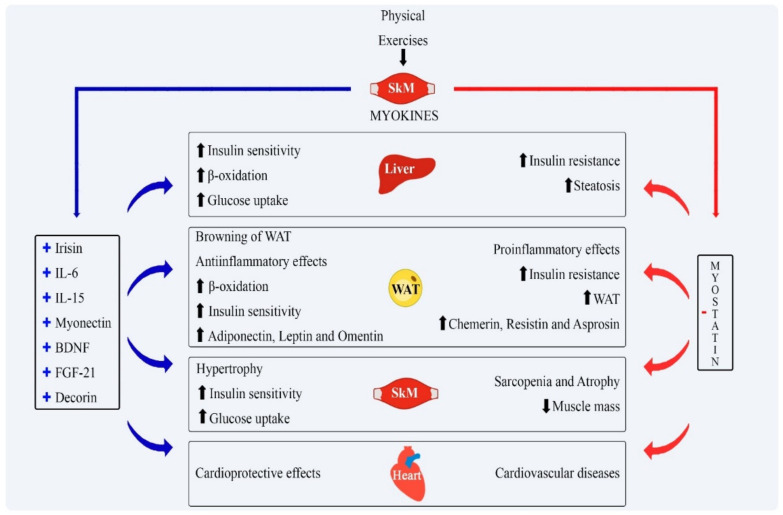
General actions of the main myokines in the metabolism. Physical exercise is the stimulus for the release of most myokines, while it inhibits myostatin release. The actions of these myokines are outlined as shown in the figure above. + represents activation; − represents inhibition; blue arrows reflect the actions of myokines stimulated by exercise; red arrows reflect the actions of myostatin; black arrows represent stimulation of physical exercises on skeletal muscle and the release of myokines. IL-6: interleukin-6; IL-15: interleukin-15; BDNF: brain-derived neurotrophic factor; FGF-21: fibroblast growth factor-21; SkM: Skeletal Muscle; WAT: White adipose tissue.

Meteorin-like protein (METRNL) is also positively regulated after resistance exercises, performing a series of beneficial actions for the metabolism of different tissues. This myokine has been shown to contribute to the browning of WAT and, consequently, to greater energy expenditure through the oxidation of glucose and FFA and reduction in fat mass [11]. METRNL also shows an anti-inflammatory role, especially in reducing adipose inflammation, increasing the expression of M2 macrophages in adipose tissue, and improving insulin resistance typical of obesity [62]. Figure 2 shows some aspects of myokines and the metabolic effects.

Decorin is a small, leucine-rich proteoglycan codificated by the decorin gene related to collagen-fibril assembly. It can bind to several cell surface receptors, is related with the modulation of tumor suppression, stimulates inflammation and autophagy and, can inhibit angiogenesis and tumorigenesis. It is secreted by skeletal muscle cells and is involved in the skeletal muscle hypertrophy and regulates muscle growth with the practice of physical exercise [63].

### 2.3. Hepatokines

Hepatokines are proteins produced by the liver that have recently been discovered as new hormones, which can worsen or improve metabolic conditions [64]. Such situations are regulated via autocrine, paracrine, and endocrine both in the liver and other tissues [18]. Even so, there is a greater interference of hepatokines in the fatty and striated skeletal muscle tissue, showing an endocrine-dependent relationship, therefore acting through crosstalk with the cytokines released by these respective tissues [14]. Table 3 brings the role of some hepatokines.

Fetuin-A is a glycoprotein mainly expressed by the liver, and its coding gene is AHSG, which is found on chromosome 3q27, this locus being typical of obesity, insulin resistance, DM2, metabolic syndrome, and nonalcoholic fatty liver disease (NAFLD) [59]. This metabolic pattern is caused due to the concentration of fetuin-A interfering with the insulin signaling cascade and the translocation of GLUT4 in insulin target tissues [79]. Fetuin-A performs deleterious functions in these metabolic diseases precisely because it can develop injury to pancreatic β cells, which are responsible for insulin production [65]. On the other hand, the decrease in fetuin-A levels by physical activity can show a beneficial potential by reducing visceral adipose tissue via a reduction in the available FFA. This allows them to capture triglycerides and add them to the white adipose tissue, therefore indirectly reducing the insulin resistance. Thus, it is still noteworthy that the reduction in fetuin-A levels can control the accumulation of visceral adipose tissue by decreasing the activation of macrophage 1, which has a pro-inflammatory action [11].

Like fetuin-A, fetuin B is the second member of the fetuin family, produced primarily by the liver and acts by inhibiting the tyrosine kinase insulin receptor. Thus, fetuin B can promote insulin resistance in myotubes and hepatocytes [18]. In humans, plasma levels of fetuin-B are elevated in obese people with fatty liver and DM2, and studies have shown that steatosis is more closely related to the development of insulin resistance than obesity itself. Indeed, it has been shown that fetuin-B decreases insulin sensitivity in muscle and hepatocytes in culture when administered to them in physiological concentrations, and, in this situation, it was not able to activate pro-inflammatory signaling [66].

Adropin is a small peptide whose concentrations are immediately regulated by the energy status and nutritional content of the diet. Studies have shown that in mice, plasma levels of adropin increase with feeding and decrease with fasting. As a result, these low levels are observed in people with obesity, polycystic ovary syndrome, fatty liver, insulin resistance, and CVD, suggesting that low levels of adropin are closely associated with metabolic disorders. On the other hand, it has been seen that in mice, the overexpression of adropin reduces weight gain, attenuates hepatosteatosis, increases fatty acid oxidation throughout the body, improves insulin sensitivity, and improves glucose tolerance [67]. A study of patients who underwent bariatric surgery showed that weight loss increased levels of adropin [80]. Still, in this respect, there is increasing evidence that adropin is a biomarker of cardiovascular dysfunction, initially because in vitro tests showed that endothelial cells treated with adropin had greater proliferation, migration, formation of a capillary-like tube, and positive regulation of the expression of nitric oxide endothelial synthase (eNOS). A recent study of patients undergoing coronary artery bypass graft surgery showed that the lowest level of plasma adropin was associated with late saphenous vein graft occlusion. Thus, it ended up providing evidence that adropin affected the progression of vascular atherosclerosis [81].

Activin E is a peptide encoded by the βE (Inhbe) inhibin gene, a member of the TGF-β superfamily, and it is predominantly expressed in the liver. A study in mice showed that activin E has several functions: initially, it is needed to induce thermogenesis by stimulating beige adipocytes in WAT; then, its overexpression raises thermogenesis and insulin sensitivity by stimulating activity of brown adipocytes in BAT; finally, activin E is closely related to FGF21 in WAT, which may increase its expression in brown pre-adipocyte culture. It is inferred that activin E may be a therapeutic target for obesity [68]. However, in humans, activin E has been reported to reduce lipolysis and increase fat accumulation in adipocytes. This is because the silencing of Inhbe’s liver expression results in increased use of total body fat in addition to decreased fat mass [69].

Sex hormone-binding globulin (SHBG) is a protein produced mainly in the liver that binds and transports sex steroids to its target tissues, and the gene that encodes it is located in the p12-p13 region of chromosome 17. At first, an association of this pathology with a low serum level of SHBG was found in post-menopausal women with NAFLD [70]. Furthermore, this low concentration has also been identified in DM2 and obese patients. On the other hand, modifications in lifestyle, such as therapy for obesity and weight reduction, caused an increase in circulating SHBG. It was recently found that adiponectin increases the production of SHBG by activating AMPK and reducing the activity of enzymes involved in liver lipogenesis. However, the relationship between SHBG and increased insulin sensitivity is not yet fully understood [71].

Chemerin is not only an adipokine, but is also produced by the liver and contributes to the decrease in glucose homeostasis. It has been found that the Paigen diet is associated with nonalcoholic fatty liver disease due to the high cholesterol content that promotes liver chemerin expression. That is, chemerin hyperexpression is found in patients with NAFLD and nonalcoholic steatohepatitis [14]. High levels of chemerin are detected in obesity, metabolic syndrome, insulin resistance, and dyslipidemia [72].

The seleno-protein is encoded by the Sepp1 gene, being responsible for transporting selenium from the liver to extrahepatic tissues [18]. When seleno-protein is found in high serum levels, it is associated with metabolic diseases, insulin resistance, and increased ROS via the activation of AMPK, and it influences the drop in concentrations of irisin, meteorin-like (METRNL), and β-aminoisobutyric acid (BAIBA), which consequently attenuates the loss of fat mass induced by physical exercises [11]. In addition, studies have shown that intermittent hypoxia generates stress that increases the levels of seleno-protein P in human hepatocytes, increasing insulin resistance, accumulating fat in adipose tissue, and aggravating risks of developing atherosclerosis [67]. Therefore, this liver cytokine plays a deleterious role when at high levels in patients with pre-diabetes and DM2, causing insulin resistance and hyperglycemia [73].

Follistatin originates from the liver and rises when the glucagon-to-insulin ratio increases in the case of fasting and aerobic and resistance exercise [74]. Patients with obesity-related to insulin resistance show an increase in the basal levels of follistatin. However, when caused by physical activity, the liver release is reduced in these situations [82]. Follistatin is able to contribute to skeletal muscle fiber hypertrophy due to its affinity for myostatin, neutralizing it and increasing skeletal muscle mass, and inducing the expression of thermogenic genes in murine adipocytes. In addition, studies with mice have shown that follistatin overexpression is associated with insulin resistance in WAT, elevated hepatic glucose production, and glucose intolerance [11]. However, after exercise, the increase in follistatin levels is beneficial since it favors successive glucose uptake by skeletal muscle tissue [83].

FGF21 is also a hepatokine and has several beneficial functions for human metabolism. Initially, it acts on insulin sensitization, increases the oxidation of fatty acids in the liver, decreases the production of glucose, increases the turnover of FFA, and reduces the development of steatohepatitis. As suggested previously, in the adipose tissue, FGF21 decreases lipolysis in vivo and in vitro, incites glucose retention in human adipocytes by inducing the expression of GLUT1 (which in fact improves insulin sensitivity in adipose tissue), increases expression and production of adiponectin, and “darkens” the white adipose tissue, turning it into brown. Thus, BAT is also affected by FGF21 in the regulation of thermogenesis [57,75]. Beyond that, studies in rats, monkeys, and humans have shown that FGF21 moderates simple sugar intake and preferences for sweet foods via signaling with FGF21 receptors in the paraventricular nucleus of the hypothalamus and correlates with decreased dopamine neurotransmission within the nucleus accumbens [84].

Angiopoietin-like 4 (ANGPTL4) is released by the liver into the circulation, especially during physical exercise because of the increased glucagon/insulin ratio, and it inhibits pancreatic lipases, which consequently decreases the absorption of fats [11]. This hepatokine also acts on WAT, stimulating lipolysis and decreasing the enzyme lipoprotein lipase (LPL) action, that is, reducing the hydrolysis of triglycerides in circulating lipoproteins to FFA and reducing the accumulation of lipids in the white tissue adipose [75]. However, the increase in serum ANGPTL4 may be related to insulin resistance in patients with metabolic syndrome compared to normal patients [70]. In this sense, it is still observed that this protein released by the liver may be entangled with other pathological processes, such as inflammation, hematopoietic stem cell activity, and invasion of cancer cells [85].

Leukocyte cell-derived chemotaxin 2 (LECT2) is a neutrophil chemotactic protein, encoded by the LECT2 gene and expressed by the liver [86]. Obese rats submitted to a diet-induced weight cycling demonstrated a high hepatic production of LECT2 [87]. Thus, it has been shown that elevated LECT2 is associated with metabolic stress, which impairs insulin signal transduction, has a responsibility to promote and express adhesion molecules, and increases the appearance of pro-inflammatory cytokines. Therefore, LECT2 can be an important marker to explain how the fatty liver leads to insulin resistance in obesity since its deletion of LECT2 increases insulin sensitivity in skeletal muscle in rats [67].

Hepassocin is a specific liver growth factor that participates in regulating hepatocyte proliferation and liver regeneration [67]. However, it has been shown that in humans, serum levels of hepassocin are elevated in pre-diabetes, DM2, and NAFLD because of their association with impaired fasting glucose, glucose intolerance, and insulin resistance [77]. Therefore, hepassocin can be a relevant biomarker for analyzing risk states for diabetes and may be known by other names, such as fibrinogen-like protein 1 and hepatocyte-derived fibrinogen-related protein 1 (HFREP1) [18].

There are still other described hepatokines, such as tsukushi and ANGLPTL8, that were described in animal models but the effects on humans are not well established. Tsukushi representes an inducible hepatokine associated with the regulation of energy expenditure and is mightily associated with obesity, nonalcoholic fatty liver disease (NAFLD), and nonalcoholic steatohepatitis (NASH). It has actions in the regulation of developmental processes in several organisms. Tsukushi regulates the expenditure of energy in part using brown fat sympathetic innervation. Hepatic and plasma levels of this hepatokine are strongly stimulated by stimuli that augment thermogenesis and energy expenditure (including adrenergic agonists and cold exposure) [78,88,89].

**Figure 3:**
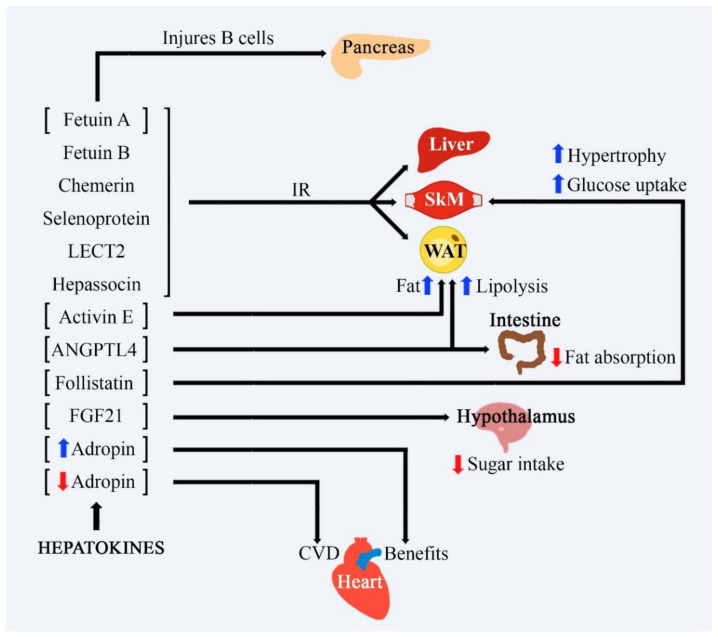
Main functions of hepatokines and their respective activities. [↓] represents the decrease and [↑] represents the increase; black arrows represent the release of hepatokines and their actions on each specified target. FGF-21: fibroblast growth factor-21; ANGPTL4: Angiopoietin-Like 4; LECT2: Leukocyte cell-derived chemotaxin 2; SkM: Skeletal Muscle; WAT: white adipose tissue; CVD: Cardiovascular disease e IR: Insulin Resistance.

Angiopoietin-like proteins (ANGPTL) constitute a group of glycoproteins that play important role as regulators of lipid metabolism. Members of the ANGPTL family have actions as regulators of LPL activity in WAT. ANGPTL3 is expressed and release by the liver and works as a regulator of plasma triglyceride levels, positively associated with glycemia, and insulin levels in patients with insulin resistance. In humans and mice, ANGPTL4 is the most studied and was discussed above. ANGPTL6 is highly expressed in obesity, augmented lipid accumulation in skeletal muscle and liver, reduced energy expenditure, and increased insulin resistance. On the other hand, overexpression of this organokine results in insulin sensitivity and elevated energy expenditure. ANGLPT8 is associated with diurnal light-dark cycle resets the master clock. Timed intake of food is also a potent synchronizer of peripheral clocks in mammals. Angptl8 is a hepatokine related to the reset of diurnal rhythms of hepatic clock, and metabolic genes in mice [88,90,91]. Figure 3 shows the main effects of the hepatokines.

### 2.4. Adipokines, Myokines and Hepatokines: Crosstalk and Metabolic Repercussions

Far beyond understanding each adipokine, myokine, or hepatokine’s actions alone, it is important to underline that these cytokines act together in the body, forming a complex network of actions in different tissues, which may have beneficial or non-beneficial effects on the genesis of various physiological disorders, and their respective outcomes, such as DM2, obesity, metabolic syndrome, and CVD [10]. Understanding this crosstalk and the subsequent pathophysiological mechanisms is valid for establishing future preventive and therapeutic measures to better manage the involved complications [59].

Present at the origin of several metabolic disorders, myostatin has negative effects on metabolism, and its levels are increased with physical inactivity and high levels of fat mass. Thus, myostatin is elevated in pro-inflammatory environments, sharing space with pro-inflammatory organokines such as TNF-α, IL-1, resistin, chemerin, and others [92]. Its contribution to resistance to the action of insulin occurs due to the decrease in phosphorylation of insulin receptor, compromising the entire insulin signaling pathway necessary to trigger its cellular actions [11]. Thus, the uptake of glucose by insulin-dependent tissues, such as skeletal muscle and adipose tissue, becomes impaired, promoting the state of hyperglycemia and allowing tissues such as those of the liver to capture the excess of glucose that will later be accumulated in the form of fat, characterizing hepatic steatosis [45].

In parallel, myostatin promotes the state of obesity, sarcopenia, and sarcopenic obesity, which promotes the release of inflammatory adipokines and hepatokines while inhibiting the release of myokines and other anti-inflammatory and antioxidant organokines [14]. Thus, myostatin, TNF-α, leptin, resistin, chemerin, fetuin-A, and other cytokines build an inflammatory and oxidative stress scenario from the polarization of macrophages (M ϕ) M1 in adipose tissue, production of reactive oxygen species, decreased insulin action, and release of FFA in plasma [11]. The hemodynamic pattern and vascular endothelial function are also altered with the pro-inflammatory secretory pattern. These cytokines, especially TNF-α and leptin, seem to aggravate hypertension, which, together with visceral obesity, dyslipidemia, and hyperglycemia, configure the metabolic syndrome scenario [59,93].

There is a wide range of disturbances in glucose and lipid homeostasis. Oxidative stress and the inflammatory state act overtime, promoting endothelial dysfunction. Hyperglycemia contributes to the production of advanced glycation end products (AGES), which, together with reactive oxygen species, cause endothelial damage, while adhesion molecules and immune cells are attracted to the vascular bed [94]. This environment constitutes fertile soil for atherosclerosis’s chronic inflammatory process, a prelude to several cardiovascular conditions [8]. Therefore, it is worth noting that myostatin has a role as a therapeutic target for managing these aforementioned metabolic disorders [45]. Many studies point to a decrease in myostatin through physical exercise, with positive effects in combating insulin resistance and reducing fat accumulation and muscle hypertrophy [95].

An important contribution to excess adiposity and favoring insulin resistance is the deregulation of the secretion and production of adipokines and myokines [96]. Adipo-myokines, such as IL-6 and TNF-α, secreted from muscle cells and adipocytes, if chronically elevated, can induce insulin resistance and consequently other comorbidities [92]. In obese and diabetic individuals, visceral fat is related to the expression of IL-6 and TNF-α produced by macrophages that prevent or attenuate insulin signaling in insulin-dependent tissues. Despite this, IL-6 released during physical exercise can improve glucose and lipid metabolism, while plasma levels of TNF-α in exercise are reduced, although its expression in adipose tissue is not affected [14].

In addition to being involved in disease metabolism, adipokines can modulate bone mineral density and bone turnover as well as skeletal muscle catabolism in aging [97]. In muscle aging, increased plasma levels of IL-6 and TNF-α, associated with loss of muscle strength, activate cell signaling pathways that lead to skeletal muscle atrophy, which in turn induce resistance to IL-6, a condition that shares similarities with insulin or leptin resistance [98]. In obesity, leptin resistance caused by hyperleptinemia can limit muscle fatty acid oxidation and reduce lipolysis of adipose tissue. This effect is neutralized with physical exercise and aggravated by an unhealthy lifestyle, as the pro-inflammatory state results in crosstalk that exacerbates the disease’s progression [99,100].

For these reasons, physical exercise is a powerful ally in the prevention and improvement of the prognosis of cardiovascular diseases, DM2, and obesity, since it influences the predominance of the secretion of anti-inflammatory myokines, adipokines, and hepatokines, such as irisin, IL-6, IL -15, myonectin, adiponectin, FGF21 (which mitigate the harmful effects of myostatin), TNF-α, resistin, chemerin, fetuin-A, and other cytokines [101]. Together, these cytokines induced in a healthy environment optimize the secretion and action of insulin; favor energy expenditure; reduce the storage of visceral fat; and, consequently, stimulate polarization of MϕM2 and reduction in Toll-like receptors (TLRs), decreasing the inflammatory pattern [11,102]. Beyond that, these cytokines appear to exhibit a protective effect on the endothelium and vascular smooth muscle, preventing atherosclerosis [103].

Studies indicate that the disproportionate release of pro-inflammatory adipokines and the reduction in lipolysis are simultaneous with insulin resistance, which plays a substantial role in the development of DM2. Insulin suppression leads to an increase in plasma fatty acid and consequent absorption by liver and muscle tissue, creating intracellular lipotoxic environments that prevent the translocation of GLUT4 in skeletal muscles and adipose tissue [104,105]. Substances that act as adipokines, myokines, and hepatokines, such as FGF21, are essential for controlling glucose and lipid metabolism. FGF21 controls insulin sensitivity by improving fat oxidation in the muscle, de novo lipogenesis (DNL) in the liver, and thermogenesis in WAT and BAT. As such, it is an important therapeutic target of protection against muscle and liver insulin resistance induced by lipids and DM2 [106,107].

It is well known that obesity, insulin resistance, and inflammation are risk factors for several metabolic disorders [108]. Overweight individuals secrete more pro-inflammatory adipokines, leading to an impaired immune response and greater susceptibility to inflammatory and infectious diseases [109]. Elevated levels of leptin and chemerin, for example, may be negatively associated with cardiometabolic health, just as adiponectin and omentin-1 may be positively associated. Therefore, the assessment of circulating adipokines may be relevant in the analysis of cardiometabolic risk [110].

In the same way that FGF21 is classified as myokine and adipokine, when working as an hepatokine, it acts as a beta-oxidation regulator and decreases lipogenesis in the liver, especially when it increases in the post-exercise period [11]. In clinical studies, patients who had DM 2 were treated with concentrations of FGF21 (with the analog LY2405319), and they showed a reduction in low-density lipoprotein (LDL) cholesterol and triglycerides, an increase in high-density lipoprotein (HDL) cholesterol, and an improvement in insulin during periods of fasting [75]. It was also shown that they identified a less atherogenic apolipoprotein profile, reducing cardiovascular risks and promoting improvements in body weight and adiponectin levels [67].

When activated by exercise, follistatin inactivates myostatin and, therefore, inhibits its pro-inflammatory effects, consequently cooperating with lipolysis in WAT. It has also been identified that follistatin directly protects β cells when induced by exercise, together with irisin [11]. However, circulating follistatin was elevated in patients with DM2 and generally correlates with insulin resistance markers, such as fasting glucose, glycated hemoglobin, and 2-h glucose during an oral glucose tolerance test [111].

Subsequently, the hepatokine most positively associated with CVD is adropin. It is expressed in endothelial cells and promotes essential functions, such as proliferation, migration, and formation of the capillary tube, together with attenuation of vascular permeability and TNF-α-induced apoptosis. Therefore, adropin is considered a protective factor of the endothelium, which increases the synthesis of nitric oxide (NO) due to the activation of eNOS. As a result, plasma levels of adropin increase with physical training and culminate with the suppression of atherosclerosis due to the regulation of monocyte differentiation, precisely its anti-inflammatory type. In this scenario, it was also observed that adropin increases fibronectin and elastin expression in vascular smooth muscle cells (VSMCs) by overloading the PI3K-Akt pathway, thus assuming that adropin regulates plaque stability and elasticity vascular [112].

On the other hand, a case–control study showed that adropin levels decreased in patients with NASH and were negatively related to serum levels of alanine transaminase, aspartate aminotransferase, and gamma glutamyl transpeptidase. It is also negatively related to the pathological changes in NAFLD, suggesting that the lower adropin expression may play an important role in the progression of NAFLD to severe NASH. Finally, another factor that implies a reduction in adropin is the oxygen-relative species, which can also culminate in NAFLD progression [113].

The main hepatokines related to insulin resistance, a fundamental factor in the development of DM2 and obesity, are fetuin-A, hepassocin, LECT2, and selenoprotein. They are usually elevated when associated with these pathologies and contribute to a state of systemic inflammation [88]. In addition to insulin resistance, fetuin-A can also promote lipotoxicity in β cells through the TLR4-JNK-NF-kB signaling pathway [65]. Finally, fetuin-B also negatively impacts metabolism because its serum level is higher in patients with coronary artery disease, especially in those with the acute coronary syndrome, and it still positively correlates with LDL-c levels [114].

**Figure 4:**
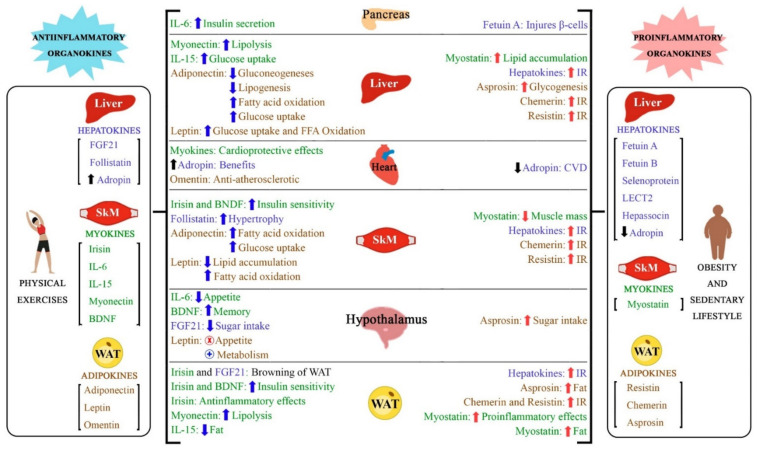
An overview of the crosstalk between adipokines, myokines and hepatokines. Adipokines are represented in brown, myokines in green and hepatokines in blue. The arrows that are indicating upward indicate an increase and downward decrease. (x) represents inhibition and (+) activation. Blue arrows are associated with benefits, while red arrows are associated with harm aspects. IL: interleukin; BDNF: brain-derived neurotrophic factor; FGF-21: fibroblast growth factor-21; SkM: skeletal muscle; WAT: white adipose tissue; CVD: cardiovascular disease; LECT2: leukocyte cell-derived chemotaxin 2; FFA: free fatty acid.

Figure 4 shows some associations among adipokines, myokines and hepatokines.

## 3. Methods

This descriptive review used the PUBMED-Medline, Embase, and Cochrane databases. The mesh terms were adipokines, myokines, hepatokines, abdominal fat, insulin resistance, obesity, type 2 diabetes, metabolic syndrome, and cardiovascular diseases. The inclusion and exclusion of the studies were based on those that included crosstalk among the organokines and not just an individual assessment of each one on insulin resistance, diabetes, obesity, metabolic syndrome, and cardiovascular complications.

## 4. Conclusions

The adipose, muscular, and hepatic tissues release several molecules that communicate through crosstalk and are capable of being biomarkers of homeostatic disorders, and they can play roles as potential therapeutic targets that can assist in methods of diagnosing, prognosis, and perhaps future treatments of metabolic syndrome and CVD. However, in vivo experimental studies in this area are further needed, addressing the crosstalk between adipokines, myokines, and hepatokines, thereby being able to support future clinical treatments.

The understanding of how myokines, adipokines, and hepatokines interact can open up new paths in addressing metabolic problems, such as resistance to the action of insulin and the development of diabetes, obesity, metabolic syndrome, and its countless repercussions in the body, such as cardiovascular diseases. The release and complex interaction of these organokines depend on numerous factors, such as the sedentary lifestyle and obesity. In this sense, the released organokines lead to numerous metabolic and physiological effects that can be harmful or beneficial to the body. They can assist in the prevention of diseases and, on the other hand, they can exert effects related to the upregulation of a pro-inflammatory state, leading to incalculable damage to the human organism.

Biochemical mechanisms of action of organokines are needed to guide future clinical trials and reduce the global burden of metabolic diseases.

## Figures and Tables

**Table 1:** Main characteristics of the adipokines.

Adipokine	Stimulation for Its Increase	Metabolic Action	Reference
Leptin	Increase in fat mass.	In the immune system, it acts to increase pro-inflammatory cytokines. In the CNS, it promotes a decrease in food intake and an increase in global energy expenditure. In skeletal muscle, it acts in the absorption and oxidation of glucose and FFA. In the liver it increases the oxidation of fatty acids and reduces the accumulation of lipids.	[11]
Adiponectina	Adrenergic beta signaling; increase in FGF21, IL-15, and irisin induced by physical exercise.	In the immune system it has anti-inflammatory actions. In the CNS it promotes an increase in food intake and a reduction in hypothalamic inflammation. In the liver and skeletal muscle, it increases fatty acid oxidation and insulin sensitivity.	[11]
Resistin	Increase in fat mass.	Immune system: pro-inflammatory actions. It acts in endothelial dysfunction, CVD and inhibition of insulin signaling through the suppressor of cytokine signaling 3 (SOCS3).	[14,15]
IL-6	Activation of the nuclear factor κ-light-chain-enhancer of activated B cells (NF-κB).	Adipose tissue: proinflammatory action, and acts in inhibiting the expression of insulin receptor substrate 1 (IRS1) and glucose transporter type 4 GLUT4) in adipocytes.	[14]
Asprosin	Induced by fasting and produced by white adipose tissue in obese people with DM2.	It increases food consumption and body weight and accelerates the production of liver glucose.	[16,17]
Chemerin	Inflammatory and coagulation serine proteases.	It accentuates glucose intolerance and makes insulin signaling difficult.	[18]
Omentin	Increase in FGF21 and dexamethasone.	Optimizes the action of insulin and, consequently, the absorption of glucose. It also acts as an anti-atherosclerotic factor.	[19]
FGF21	Exposure to cold and physical exercise.	It acts in the browning of WAT, lipid oxidation and thermogenesis, and stimulates the expression of adiponectin in the bloodstream.	[20]
SFRP5	Induced during the proliferation, differentiation and maturation of pre-adipocytes.	Regulates the expression of pro-inflammatory cytokines by inhibiting the Wingless-type family member 5a signaling (Wnt5a), non-canonical Wnt family.	[14,21]
Lipocalin 2	Low-level systemic inflammation in obese patients with metabolic syndrome.	Regulation of inflammation and the transport of fatty acids and iron. It is associated with CVD, vascular remodeling and instability of atherosclerotic plaques.	[22,23]
Vaspin	Increase in fat mass.	Reduces the synthesis of pro-inflammatory cytokines. It improves glucose intolerance and insulin sensitivity and protects the vascular tissues from fatty acid-induced apoptosis.	[24,25]
FSTL1	It is expressed in large quantities by adipose tissue in the state of low-grade chronic inflammation.	Lower levels of FSTL1 are associated with super obesity due to loss of adipogenesis, increased maturated adipocytes, cellular senescence and anti-apoptotic FSLT1 reduction. It has a pro-inflammatory action and possible relationship with overweight and obesity.	[26]
Sparc	Secreted by adipocytes, promoted adipose tissue fibrosis and inhibited adipogenesis.	Responsible for modulating the expression of pro-inflammatory cytokines that act on insulin resistance; and inhibits adipogenesis.	[23,27]
CTRPs	Expressed in conditions of adiponectin and leptin deficiency and high body mass index.	Regulation of inflammatory processes of adipose tissue. Regulation of glucose and fat metabolism in peripheral tissues and food intake.	[23,28]
FAM19A5	Increase in fat mass.	Inhibits the proliferation and inflammation of vascular smooth muscle cell related to cardiovascular diseases through obesity.	[29]
WISP1	Obesity, adipogenesis and visceral fat abnormalities.	Stimulates the cytokine response in macrophages associated with tissue adipose; induces the proliferation of mesenchymal stem cells, which increases tissue adipose.	[30]
Progranulin	Increase in fat mass associated with obesidade visceral, DM2 and dislipidemia.	It has anti-inflammatory properties. Hyper-progranulinemia is associated with insulin resistance and deficient insulin signaling.	[31,32]
Nesfatin-1	Unclear	Induces satiety, which promotes body weight reduction. It can also regulate gastric distension and motility via the melanocortin pathway in the central nucleus of amygdala.	[23]
Visfatin	Increase in fat mass.	It produces adipocyte inflammation, insulin resistance and pancreatic beta cell dysfunction.	[33,34]
Fetuin-A	Increase in fat mass.	Associated to insulin resistance and inflammation.	[35]
ZAG	PPARγ, glucocorticoids, certain β3-adrenergic receptor agonists, thyroid hormones, and growth hormone (GH).	It acts in the acceleration of lipid metabolism, regulating enzymes of lipogenesis and lipolysis and stimulating production of adiponectin and BAT.	[36,37,38]

BAT: brown adipose tissue; CNS: central nervous system; CTRPs: C1q/TNF-related proteins; CVD: cardiovascular disease; DM2: diabetes mellitus type 2; FAM19A5: family with seuence similarity to 19 member A5; FFA: free fatty acid; FGF21: fibroblast growth factor 21; FSTL1: follistatin-like 1; IL: interleukin; SFRP5: secreted frizzled-related protein 5; Sparc: secreted protein acidic and rich in cysteine; TNF-α: tumor necrosis factor-alpha; ZAG: zinc-α2-glycoprotein; WAT: white adipose tissue; WISP1: wingless-type inducible signaling pathway protein-1.

**Table 2:** Main characteristics of the myokines.

Myokine	Stimulation for Its Increase	Metabolic Action	Reference
Irisin	Physical exercise.	Darkening of WAT, increases energy expenditure, improves insulin sensitivity and induces weight loss.	[8]
BAIBA	Aerobic exercise	It acts in the Browning of adipose tissue, lipid oxidation and reduces insulin resistance.	[11]
Myostatin	Sedentary lifestyle	Induces muscle mass loss associated with insulin resistance and fat accumulation in the liver. Facilitates body fat accumulation.	[45]
Follistatin	Expressed in the context of physical activity, especially aerobic, resistance or high intensity training.	Inhibits the actions of myostatin, contributing to hypertrophy of skeletal muscle and reduction in fat mass, with consequent optimization of glucose uptake.	[13]
FGF21	Physical exercise.	Increases insulin sensitivity, reduces plasma glucose and acts on lipolysis.	[57]
Apelin	Resistance exercises.	Anti-inflammatory role. It acts in the formation of new vessels and in the control of cardiac muscles and blood pressure.	[58]
Myonectin	Resistance exercises.	Increases the uptake of lipids by adipose tissue and liver, decreasing the plasma concentration of FFA.	[20]
IL-6	Physical exercise.	Pro-inflammatory cytokine associated with insulin resistance in obesity.	[59]
IL-15	Released after acute episodes of aerobic exercise.	Anti-inflammatory properties by inhibiting TNF-α expression; contributes to muscle hypertrophy, reduction in visceral adipose tissue and optimizes insulin action.	[14]
Sparc	Resistance exercises and muscle hypertrophy.	Inhibition of adipose tissue formation, increased insulin release and optimization of glucose uptake.	[60]
BDNF	Muscle and brain induced after exercise.	When produced by muscle, it increases sensitivity to insulin.	[61]
METRNL	Resistance exercises.	Anti-inflammatory role. Contributes to the browning of WAT and energy expenditure through the oxidation of glucose and FFA.	[62]
Decorin	Expressed in response to acute or chronic endurance training	Binds to myostatin by inhibiting its actions. As a consequence, it induces hypertrophy in the skeletal muscle.	[63]

BAIBA: β-aminoisobutyric acid; BDNF: brain-derived neurotrophic factor; FFA: free fatty acid; FGF21: fibroblast growth factor 21; IL-6: interleukin 6; IL-15: interleuckn 15; METRNL: meteorin-like protein; Sparc: secreted protein acidic and rich in cysteine; TNF-α: tumor necrosis factor-α; WAT: white adipose tissue.

**Table 3:** Main characteristics of the hepatokines.

Hepatokine	Stimulation for Its Increase	Metabolic Action	Reference
Fetuin A	Related to obesity, especially NAFLD and the increase in VAT.	It causes injury to the pancreas B cells and insulin resistance and works as a predictor of DM2.	[59,65]
Fetuin B	Increased in humans with steatosis and is related to insulin resistance.	Promotes insulin resistance and the development of diabetes.	[66]
Adropin	Regulated positively with food intake and weight reduction.	Stimulates lipolysis throughout the body, reducing weight gain and hepatic steatosis, optimizing the action of insulin and preventing the progression of atherosclerosis.	[67]
Activin E	High with obesity and NAFLD.	Reduces lipolysis and increase fat accumulation in adipocytes.	[68,69]
SHBG	Weight reduction and healthy lifestyle.	Transport of sex steroids to its target tissues. The increase in insulin sensitivity, stimulated by SHBG, is not yet fully cleared.	[70,71]
Chemerin	Produced in a state of obesity, dyslipidemia, metabolic syndrome and DM2.	Impairment of glucose homeostasis, increases insulin resistance and fat accumulation in the liver.	[14,72]
Selenoprotein	Associated with metabolic diseases, insulin resistance and hypoxia.	Attenuates fat loss induced by exercise. In hypoxia, insulin resistance and fat accumulation in adipose tissue increases.	[11,73]
Folistatin	It increases when the glucagon-to-insulin ratio rises in situations of aerobic exercise and resistance.	Actions on skeletal muscle hypertrophy, which increases glucose capture, and on the expression of thermogenic genes in murine adipocytes.	[11,74]
FGF21	Aerobic exercises	It increases the sensitivity to insulin, the oxidation of fatty acids in the liver, decreases the production of glucose and the development of hepatic steatosis.	[11]
ANGPTL4	Physical exercise	Stimulates lipolysis and decreases the action of the LPL enzyme on white adipose tissue. Inhibits pancreatic lipase and consequently decreases fat absorption.	[11,75]
ANGPTL4	Food signals	Mediates food-driven resetting of circadian clock in mice liver; associated with regulation of inflammation, lipid metabolism, cancer cell invasion, and hematopoietic stem activity	[76]
LECT2	Associated with metabolic stress	Impairment of insulin signal transduction and increases the appearance of pro-inflammatory cytokines.	[67]
Hepassocin	Elevated in pre-diabetes, DM2, and NAFLD.	Participates in the regulation of hepatocyte proliferation and liver regeneration.	[67,77]
Tsukushi	In response to NAFLD.	Reduces HDL-c cholesterol; reduced cholesterol efflux capacity, and reduces cholesterol-to–bile acid conversion in the liver.	[78]

ANGPTL4: angiopoietin-like 4; DM2: diabetes mellitus typo 2; FGF21: fibroblast growth factor 21; LPL: lipoprotein lipase; LECT2: leukocyte cell-derived chemotaxin 2; NAFLD: nonalcoholic fatty liver disease; SHBG: sex hormone-binding globulin; VAT: visceral adipose tissue.

## Data Availability

NA.
